# Depth-adapted adaptive optics for three-photon microscopy

**DOI:** 10.1364/BOE.609127

**Published:** 2026-08-13

**Authors:** Qi Hu, Jingyu Wang, Huriye Atilgan, Armin Lak, Martin J. Booth

**Affiliations:** 1 Department of Engineering Science, University of Oxford, Oxford, UK; 2 Department of Physiology, Anatomy, and Genetics, University of Oxford, Oxford, UK

## Abstract

Three-photon (3-P) fluorescence microscopy enables deep in vivo imaging with subcellular resolution, but its performance is fundamentally constrained by the maximum permissible laser power required to avoid tissue heating and photodamage. Under these power-limited conditions, fluorescence signal generation, image contrast, and achievable imaging depth are strongly affected by the illumination beam profile and aberration correction strategy. In this paper, we showed that using a fixed illumination beam size was suboptimal across different imaging depths. We further showed that conventional Zernike-based adaptive optics (AO) correction degrades under reduced Gaussian illumination beam sizes due to loss of modal orthogonality. This degradation results in slow convergence, unintended focal and field-of-view shifts, and excessive wavefront deformations. To overcome these limitations, we introduced a depth-adapted AO framework in which both the illumination beam profile and the aberration correction basis were dynamically matched to the imaging conditions. By combining depth-optimised beam underfilling with a bespoke set of illumination-matched aberration modes, we achieved faster and more stable AO convergence, enhanced fluorescence signal, and image quality during deep in vivo multi-channel neuroimaging. Together, these results established a practical and robust AO-enabled three-photon microscopy strategy that enhanced imaging performance under realistic power constraints.

## 1. Introduction

Three-photon (3-P) microscopy, for its long excitation wavelength and non-linear absorption, has become one of the most promising less-intrusive in-vivo deep imaging techniques for both structural and functional studies [1–5]. In multiphoton imaging, the non-linear absorption ensures intrinsic optical sectioning, i.e. fluorescence occurs only at the focal volume. Compared to two-photon imaging, the longer wavelength light is less susceptible to scattering and allows the illumination to penetrate deeply into the tissue; the cubic dependence on excitation intensity also produces a superior signal-to-background ratio. Combined with sparse or cell-type–specific labelling, the technique becomes highly suitable for functional imaging of dynamic biological processes, including the monitoring of calcium transients using genetically encoded indicators such as GCaMP [6,7].

Despite these advantages, there are still challenges in 3-P imaging. As the illumination beam propagates deeper into the tissue, the absorption and any residual scattering significantly reduce the signal-to-background for deep focusing [8]. Illumination power exponentially decreases with the propagation distance due to dissipation, while energy absorption increases with prolonged exposure; this could lead to phototoxicity, physiological changes, and photothermal damage if the illumination power is not carefully selected, particularly where short pulsed lasers are used [7,9,10]. Therefore, optimising illumination to maximise fluorescence yield within the limits imposed by thermal damage is critical for extending the imaging capability, especially for animal imaging [11,12].

One proposed strategy to overcome these limitations is to adjust the spatial profile of the excitation beam by optimising the underfilling factor at the objective back aperture. Simulation studies have suggested that adjusting the effective illumination numerical aperture (NA) in this way can influence the trade-off between multiphoton fluorescence excitation efficiency and light attenuation [12]. A larger NA theoretically produces a tighter focal volume, but at the cost of a longer optical path due to higher propagation angles through tissue, leading to higher overall absorption. These competing effects imply that the optimal underfilling ratio depends on the imaging depth and the tissue’s optical properties. It is well known that an underfilled aperture is preferable for illumination, but a high-NA objective remains advantageous for fluorescence collection. Although several studies have implemented underfilling in multiphoton microscopy, systematic experimental demonstrations across different tissue depths are limited in scope [13].

Despite the long wavelength illumination being less susceptible to tissue scattering, aberration correction using adaptive optics (AO) remains crucial for restoring imaging quality in multi-photon microscopes [14–18]. AO compensates wavefront distortions arising from optical misalignment, sample mounting, surgically implanted optical interfaces (e.g., cranial windows), and structural inhomogeneities within the tissue, thereby significantly improving imaging performance [19–21]. In practice, many multiphoton imaging systems employ sensorless AO, in which aberrations are inferred indirectly from image-based metrics rather than measured with a dedicated wavefront sensor. Conventional sensorless methods often rely on Zernike polynomial modes for wavefront correction. A major reason for this is the modes’ orthogonality over uniformly illuminated pupils. However, their effectiveness is compromised when used in systems with underfilled illumination, which commonly produces a truncated Gaussian beam profile.

Zernike polynomials have sharp peripheral slopes; this effectively means that most modulation power concentrates close to the edges of the AO active area, where little laser energy is present due to the Gaussian beam profile. The mismatch between the Gaussian profile and the ideal uniform illumination affects orthogonality between the modes and results in slow and ineffective aberration compensation. In addition, to correct aberrations for Gaussian beams using Zernike modes where central gradients are moderate, it normally requires modes with huge coefficients for effective correction. Large modal coefficients lead to sharp corresponding peripheral gradients, which place difficult demands on AO correction elements. Although some modified Zernike polynomials have been derived that achieve perfect orthogonality under Gaussian weighting, such modes tend to have even sharper peripheral slopes, creating further difficulties for practical application [22]. This highlights the need for an alternative modal basis that is both orthogonal under Gaussian illumination and practical to implement experimentally.

In this paper, we present a novel adaptive 3-P microscopy scheme, whereby illumination profiles and aberration basis modes are adapted according to the imaging depth. This scheme is demonstrated through practical imaging in awake mouse brain cortex. We undertook a systematic experimental comparison of different illumination configurations and their effects on the imaging quality under constant total laser power at different imaging depths. We introduced a new set of orthogonal modes – weighted-Bessel modes – which are both theoretically orthogonal and practical to use for Gaussian illumination. We compared sensorless AO performance using conventional Zernike modes and weighted-Bessel modes for correcting sample-induced aberrations when imaging deep inside awake mouse brains. Weighted-Bessel modes optimised for a specific imaging depth were observed to have more robust correction, with faster and more stable convergence during those imaging events.

## 2. Methods

### 2.1. Three-photon adaptive optics microscope

**Fig. 1.**
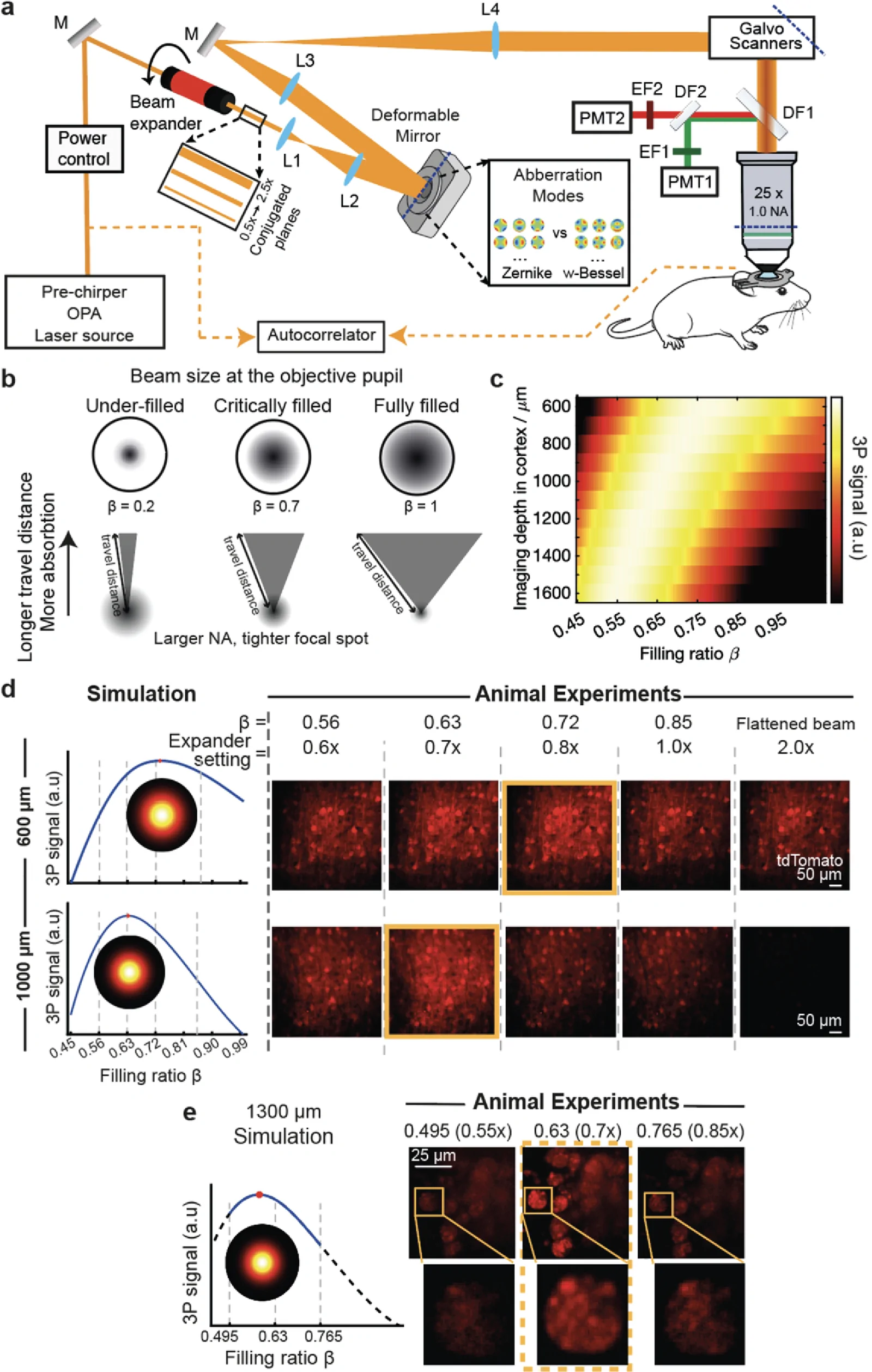
**Depth-dependent adaptation of the illumination beam profile in three-photon microscopy.** (a) Schematic of the adaptive three-photon microscope, incorporating a beam expander and a deformable mirror, for this study. (b) Conceptual illustration of under-filled, critically filled, and fully filled objective back apertures, illustrating the trade-off between focal spot size, excitation efficiency, and propagation loss as a function of the underfilling parameter β. (c) Numerical simulation predicting the optimal illumination filling ratio at the objective back aperture as a function of imaging depth. At each depth, the simulated fluorescence intensity was normalised such that the maximal intensity equals to 1. (d) In vivo three-photon images with system flat aberration correction applied acquired at 600 and 1000 μm depths in the medial frontal cortex using different illumination beam sizes (β), adjusted via the beam expander. (e) Experimental validation at 1300 μm depth with system flat pre-compensation applied, comparing simulated point-spread functions and corresponding in vivo images for different β values, confirming a further reduction in optimal beam filling at greater depths.

3-P fluorescence imaging was performed with a Cronus 3P system (Light Conversion, Lithuania) optimised for 1300 nm excitation (Fig. 1(a), See Supplement 1 for details of the system). Briefly, imaging was conducted using a galvo scanner-based multiphoton microscope with a 25× water immersion objective (1.0 NA, 4 mm WD). The tunable beam expander (Thorlabs ZBE1C) was positioned before the deformable mirror so that the conjugation and magnification between the deformable mirror, scanners, and pupil back aperture was not affected when the beam size was adjusted (Fig. 1(a)). This is because it is a common practice to ensure the conjugation relationship between the deformable mirror and the objective back aperture in an adaptive microscope to ensure an optimized adaptive optics configuration. By tuning the beam expansion, we controlled the degree of objective back-aperture filling, allowing a trade-off between focal confinement and excitation path length in tissue (Fig. 1(b)).

The illumination beam size at the objective back aperture was parameterised by the underfilling ratio β, defined as the ratio of the Gaussian beam radius (1/e^2^) to the objective back-aperture radius. For the truncated Gaussian beam profile, β was defined as the ratio of the beam radius to its edge and the objective back-aperture radius. The limiting aperture of the optical system was the deformable mirror (DM) aperture, which mapped to a β value of 0.85 (for Imagine Optic Mirao-52e or 0.77 for Alpao DM97, which was used temporarily in our system to collect results in Figure S1, S2 and S4 e-h). A beam expander allowed us to optimise the Gaussian beam profile with a continuously tunable zoom factor of 0.5× to 2.5×, corresponding to β values ranging from 0.45 to 0.85 (or 0.77 for Alpao DM). This effectively means that the maximal β value of 0.85 was achieved with a beam expansion of 0.95× (or 0.77 achieved by 0.85×); any further beam expansion flattened the beam but did not increase β.

This system configuration was designed to allow a good use of DM actuator strokes when a reduced illumination beam size was used for imaging. From the simulation results (Fig. 1(c)), the optimised underfilling ratio β for any imaging depths more than 600 μm was always below 0.75, smaller than the DM aperture size projected at the objective pupil, showing that our choice of magnification is suitable. Any further beam expansion would not lead to a higher filling ratio, as it was imposed by the limit of the DM aperture, but only produce a flattened beam profile.

### 2.2. Illumination beam size optimisation

Two opposing factors influence how a beam travels through tissue and how much 3-P signal it generates. A high-NA system focuses light more tightly to form a smaller focal volume and enhance 3-P fluorescence generation. However, the wider illumination angle leads to longer propagation paths through tissue, causing greater power loss from absorption and scattering. Using the numerical method of [12], we optimised how much the objective’s back aperture should be underfilled by a Gaussian beam in order to maximise fluorescence at different imaging depths in tissue (see the Supplement 1 for more details). Using the results of the attenuation depth measurement of different brain structures in literature [12,14], we optimized the beam profile expansion for different imaging events numerically, and we verified performance experimentally. For experimental verification, we calibrated the optical system for accurate adjustment of the illumination beam expansion (see details of calibration in Supplement 1). The average laser power, measured after the objective lens, was adjusted to maintain constant when changing the beam size for fair comparisons.

### 2.3. Weighted-Bessel (w-Bessel) mode

Many implementations of AO in microscopy use indirect wavefront sensing (sensorless) methods to correct aberrations with AO devices. Such methods normally compensate aberrations iteratively using an orthogonal set of mode bases, deriving the optimised coefficient of each modal component.

There is an inherent issue with Zernike modes when used to correct aberrations in a (truncated) Gaussian beam. The beam profile means that the orthogonality of Zernike polynomials is degraded, leading to modal cross-coupling and unstable convergence (details of this analysis are discussed in the results section). To overcome these limitations, we derived a new set of Bessel function-based modes, called weighted-Bessel (w-Bessel) modes, that are orthogonal for Gaussian beam of different sizes. Similar to Zernike modes $Z_{n}^{m}$ , the mode $B_{n}^{m}$ has a radial order of *n* and an angular order of *m*, where |m|≤n and m=n−2l such that *l* is a whole number. This should not be confused with “Bessel beams” that have a Bessel amplitude function and have also been used in 3-P imaging for extending the depth of focus (Chen, et al. 2018). We define w-Bessel modes as

(1) $$B_{n}^{m}(r,\theta )=N\;J_{\left|m\right|}\left(r\;j_{|m|,\;\frac{n-|m|}{2}+1\;}\right)\text{ang}(m\theta )\frac{1}{\sqrt{\;\hat{I}(r,\theta )}}$$

where *N* is the Zernike normalisation defined as

$$N=\{\begin{array}{ll} \sqrt{n+1} & \text{if}\;m=0\;\\ \sqrt{2n+2} & \text{if}\;m\neq 0 \end{array}$$

and ang(mθ) is the angular term defined the same as for the Zernike polynomials,

$$\text{ang}(m\theta )=\{\begin{array}{ll} 1 & \text{if}\;m=0\\ \text{sin}(-m\theta ) & \text{if}\;m<0\\ \text{cos}(m\theta ) & \text{if}\;m>0 \end{array}$$

$J_{\left|m\right|}(r)$ is the |*m*|th order Bessel function of the first type and $j_{|m|,\;\frac{n-|m|}{2}+1}$ is the $\left(\frac{n-|m|}{2}+1\right)\text{th}$ zero of the |*m*|th order Bessel function of the first type. $\hat{I}(r,\theta )$ is the normalised intensity distribution of the Gaussian illumination profile defined as $\hat{I}(r,\theta )=\frac{I(r,\theta )}{\int \;\;\;\int I(r,\theta )\;r\;dr\;d\theta }$ . *r* and *θ* are the radial coordinates where 0≤r≤1 and −π≤θ<π .

The illumination beam profile is included in the w-Bessel mode definition, similar to the approach in the derivation of Gaussian Zernike modes in [22]. W-Bessel modes are thus not a single fixed set of modes but are a class of modes adapted according to the beam expansion, which may be optimised depending on the imaging depth and the attenuation depths of different specimen tissue structures.

These modes were defined heuristically to provide the desired properties of orthogonality and reduced phase magnitude and gradient at the periphery compared to Zernike polynomials. We note that they do not form a complete set; this is readily seen as each of the modes at r=1 is set to zero. However, this is of limited practical concern, as the amplitude of the light reaching the focus at the edge of the aperture is small, hence the phase modulation at the edge has little effect on imaging. In addition, in practical modal AO systems, it is typical to include only selected low-order modes for aberration correction; the truncated correction series never consists of a complete set. The fact that the selected weighted-Bessel modes are defined to be orthogonal for a Gaussian beam, and the corresponding Zernike modes are not, means that w-B modes are better suited to correcting these aberrations. This is shown clearly in our results in the latter part of this paper.

### 2.4. Orthogonality analysis

To understand the modal independence with respect to a Gaussian illumination profile, we defined the modal orthogonality according to the inner product

(2) $$M_{i}\;,M_{j}=\frac{1}{\pi }\int \;\;\;\int M_{i}(r,\theta )M_{j}(r,\theta )\;\;\hat{I}(r,\theta )\;r\;dr\;d\theta$$

**Fig. 2.**
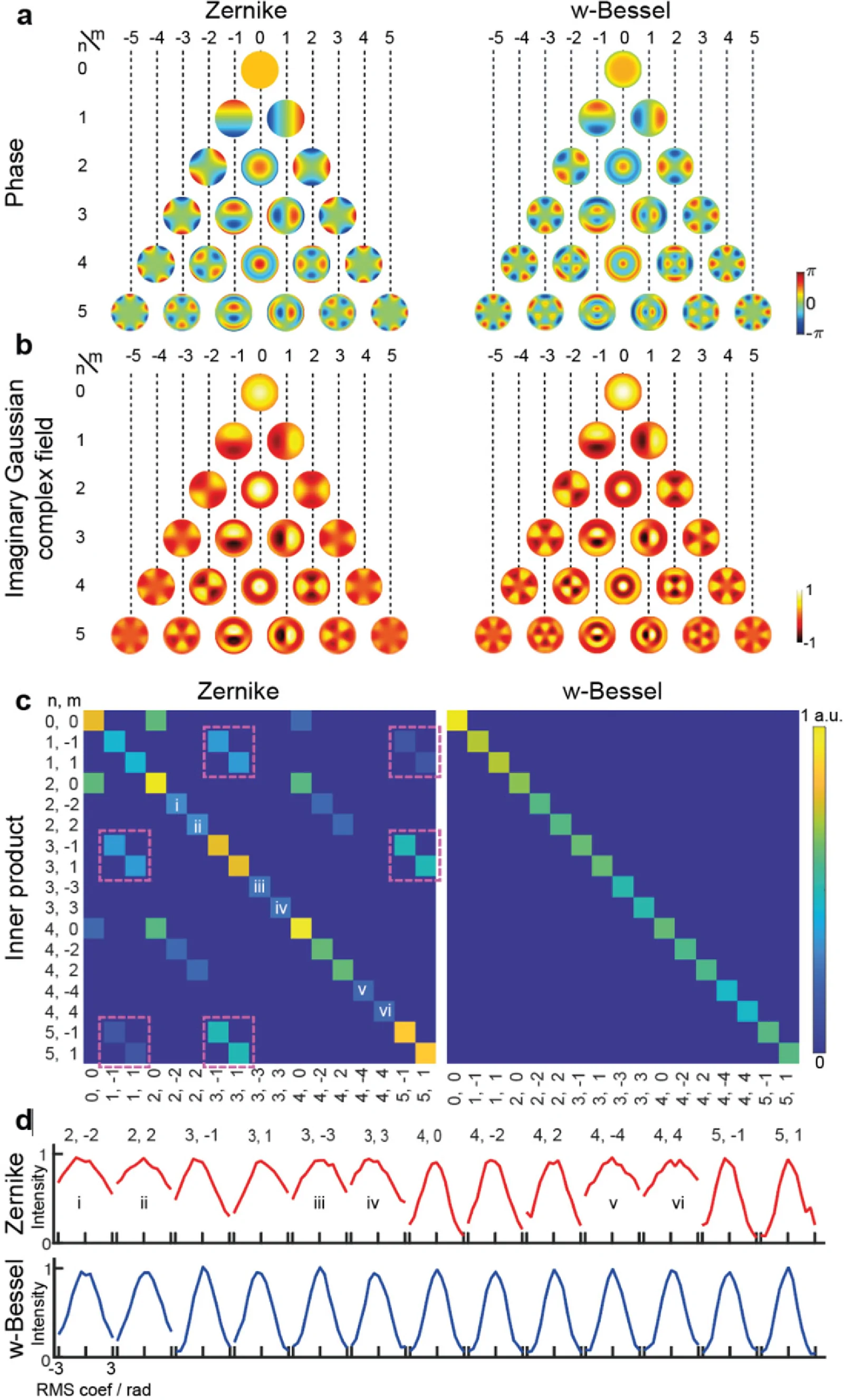
**Weighted-Bessel modes preserve orthogonality under Gaussian illumination and improve sensorless adaptive optics performance.** (a) Phase patterns of conventional Zernike (left) and weighted-Bessel (w-Bessel; right) modes, shown in the same (n,m) index ordering. (b) Corresponding imaginary part of the Gaussian-weighted complex field for each mode under underfilled illumination. (c) Inner-product matrices (Eq. (2)) computed over the Gaussian-weighted pupil, showing increased off-diagonal coupling for Zernike modes and strong diagonal dominance for w-Bessel modes. (d) Representative sensorless intensity response curves (normalised to the aberration-free intensity value) versus applied RMS mode coefficient, illustrating more consistent uni-modal responses for w-Bessel compared to Zernike. Six highlighted high angular order Zernike modes i-vi showed low inner products (in c) and compromised modal influence (in d).

Where *M_i_* and *M_j_* are the *i*th and *j*th modes of an aberration expansion set; *i* and *j* are single indices following (but not limited to) Noll’s indices. The orthogonality analysis results of the w-Bessel modes and Zernike modes are computed using the modulus of the inner product and are presented in Fig. 2(c).

## 3. Results

To optimise the 3-P fluorescence generation, we systematically analysed the effects of the beam size when imaging at different cortical depths in an awake mouse brain. We also compared the performance of the conventional Zernike polynomials with the proposed w-Bessel modes in convergence speed, stability and correction quality when used for compensating sample-induced aberrations through sensorless AO methods.

### 3.1. Depth-dependent adaptation of the illumination beam profile

We investigated, through both modelling and experiment, the trade-offs involved in choosing the optimal objective underfilling ratio for 3-P excitation in different imaging depths, as explained in the method section. We carried out numerical simulations adapted from (Wang, et al. 2015), to analyse the consequences of varying illumination conditions (Fig. 1(c)). In agreement with the previous study, the simulations suggested optimal beam profiles at the objective back aperture to maximise the fluorescence signal at different imaging depths in the mouse cortex.

For experimental validations, the illumination beam size was continuously tunable using the beam expander. To enable a fair comparison, we maintained a constant laser power after objective while adjusting the illumination beam size to compare the collected fluorescence intensity. We selected three imaging depths within the medial frontal cortex — 600, 1000, and 1300 μm — where the anatomical organisation allows continuous imaging into deeper tissue (see the Beam profile measurement section in the Supplement 1). At each depth, we imaged the same cortical field of view using different illumination beam sizes. The change in fluorescence intensity with the beam size was consistent with the simulated results (Fig. 1(d), e).

At 600 μm depth, a beam size with the underfilling parameter β≈0.72 produced the brightest images, matching the simulated optimum (β≈0.73) (Fig. 1(d), top panel). Moving deeper into the tissue, the optimised beam size progressively decreased in simulation, with β≈0.63 at 1000μm,β≈0.58 at 1300μm (Fig. 1(d), e); the trend of a decreasing optimised underfilling parameter *β* with an increasing imaging depth was observed experimentally. In addition, it was observed that beam size adjustment had a more pronounced impact on image quality at greater depths, as illustrated at 1300μm deep (Fig. 1(e)).

Overall, we confirmed experimentally that the optimal beam expansion systematically decreases with increasing imaging depth, from β≈0.73 at 600μm depth to 0.58 at 1300μm depth when imaging in the cortex. Both the simulation and experimental results showed that it was necessary to adapt the beam illumination profile with depth in order to maintain optimum imaging quality.

### 3.2. Weighted-Bessel mode and orthogonality validation

The need for depth adaptation of the illumination profile had consequences for the performance of sensorless AO aberration correction. Due to compromised modal orthogonality under Gaussian underfilling, Zernike-based correction progressively degraded as the beam size decreased. To address the issue, we investigated the properties of the new w-Bessel modes. The orthogonality with respect to the Gaussian intensity distribution was quantified using the inner product defined in Eq. (2), providing a robust foundation for evaluating performance against Zernike modes.

We evaluated the orthogonality of w-Bessel modes and Zernike modes, taking into account the measured Gaussian laser profile (β≈0.72) . As shown in Fig. 2(c), w-Bessel modes are perfectly orthogonal in simulation, indicated by the diagonal matrix; Zernike modes, on the other hand, have crosstalk among modes of the same angular order (m order), as shown by the non-zero off-diagonal components.

While the modal phase patterns differ substantially between bases (Fig. 2(a)), the focal impact of these modes is more clearly understood by examining the resulting complex field of the Gaussian illumination, where both phase and amplitude contribute. For this reason, we compare the imaginary component of the field after applying each mode (Fig. 2(b)). Under Gaussian illumination, the contribution from the pupil periphery is strongly suppressed, leading to similar imaginary field patterns for tip, tilt $\left(Z_{1}^{\pm 1}\right)$ , coma $\left(Z_{3}^{\pm 1}\right)$ and secondary coma $\left(Z_{5}^{\pm 1}\right)$ despite their distinct phase profiles. This effect is mitigated for the w-Bessel modes, which better preserve modal differentiation across the illuminated pupil.

We also analysed the intensity variations caused by aberration modes ranging from −3 to +3 rad rms through experimental measurements on a water-immersed beads sample (Fig. 2(d)). W-Bessel modes showed symmetrical intensity variations of consistent strength for all the selected low-order common modes. In contrast, Zernike modes had inconsistent modulation effects, with high m-order modes introducing only moderate modulations (Fig. 2(d); astigmatism: $Z_{2}^{\pm 2}$ , trefoil: $Z_{3}^{\pm 3}$ , quatrefoil: $Z_{4}^{\pm 4}$ ). This was also reflected in the orthogonality analysis, where the self inner product of the high m-order Zernike modes was small. In contrast, all the modes (apart from the first four low-order modes: $B_{0}^{0},B_{1}^{\pm 1}$ , and $B_{2}^{0}$ ) in the w-Bessel set had relatively consistent inner product (Fig. 2(c)).

Furthermore, the effects of Zernike coma $\left(Z_{3}^{\pm 1}\right)$ and its corresponding w-Bessel mode $\left(B_{3}^{\pm 1}\right)$ were compared when applied to a bead sample, as shown in Supplement 1 Figure S1. Results showed that $B_{3}^{\pm 1}$ introduced a larger intensity variation and little change to the centre of gravity of the beads x-y plane. This is a direct effect of the cross-talk between Zernike coma $\left(Z_{3}^{\pm 1}\right)$ and tip, tilt $\left(Z_{1}^{\pm 1}\right)$ modes. On the other hand, $Z_{4}^{0}$ introduced little intensity variation but a large axial shift, which suggested that the spherical had a strong cross-talk with the defocus mode $\left(Z_{2}^{0}\right)$ as shown in Supplement 1 Figure S2.

Modal orthogonality and field modulation have direct impacts on the convergence speed (i.e. the number of correction iterations before the calculated coefficients converge) and correction accuracy when applied to sensorless AO aberration correction. As we will show in the next section, when used for correcting aberrations in deep tissue imaging, Zernike-based correction converged much more slowly, led to extreme resultant peak-to-valley wavefront shapes and compromised imaging performance.

### 3.3. Improved convergence and stability of sensorless AO using weighted-Bessel modes

To compare the practical performance of Zernike and w-Bessel modes for sensorless AO, we performed in vivo three-photon imaging in an awake cortex of transgenic mice expressing GCaMP in CaMKII-positive neurons, with tdTomato introduced in a subset of cells for dual-colour imaging. The illumination beam size was adjusted according to imaging depth, as determined by the preceding analysis. System aberrations were first corrected using a bead sample sealed by a coverslip of 0.17mm , and the resultant correction was applied as a starting point for all subsequent in vivo experiments.

**Fig. 3.**
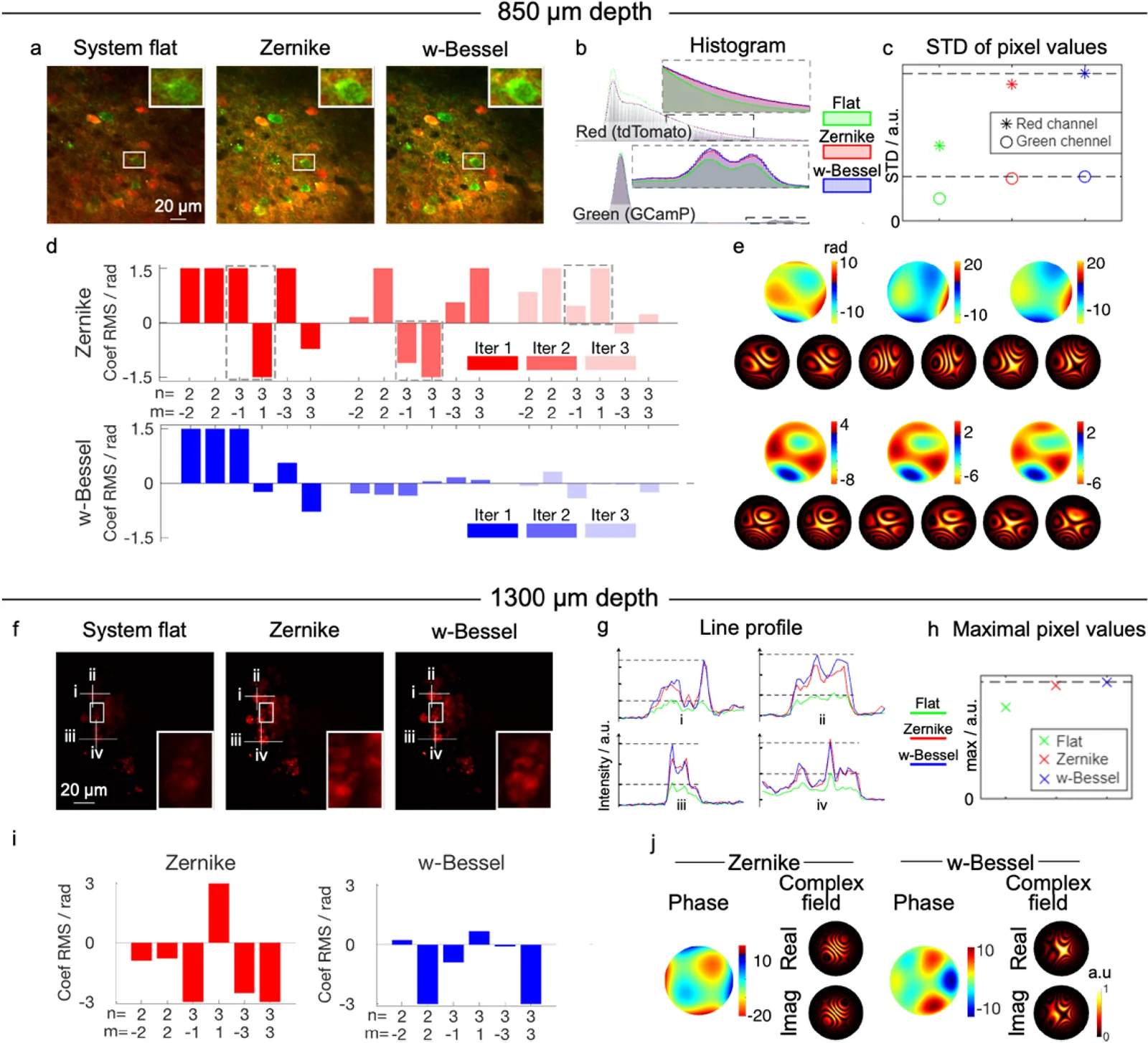
**Improved convergence, stability, and image quality using weighted-Bessel (w-Bessel) modes for sensorless adaptive optics correction in vivo.** Representative in vivo three-photon images acquired at (a) 850 μm and (f) 1300 μm depth under system-flat conditions, after Zernike- and w-Bessel-based corrections. Insets show magnified regions. Scale bars, 20 μm. (b) Intensity histograms of red (tdTomato) and green (GCaMP) channels for the three correction conditions. (c) The standard deviation (STD) of all red and green channels pixel values in the images in (a). Calculated modal coefficients (d) across three successive iterations of sensorless correction at 850 μm depth and (i) after one iteration of correction at 1300 μm depth (e) Phase maps and corresponding complex fields (real and imaginary components) reconstructed from the final wavefront corrections. (g) Line profiles extracted from the regions indicated in (f). (h) The maximal pixel values of images in (f). (j) Phase and complex intensity components of the final Zernike- and w-Bessel-corrected fields, respectively.

Imaging was performed at depths of 850μm and 1300μm from the cortical surface (Fig. 3). In each case, six low-order modes (astigmatism $Z_{2}^{\pm 2},B_{2}^{\pm 2}$ , coma $Z_{3}^{\pm 1},B_{3}^{\pm 1}$ , and trefoil $Z_{3}^{\pm 3},B_{3}^{\pm 3}$ ) were included for correction using either the Zernike or w-Bessel basis. The spherical mode was pre-compensated during the system aberration correction process using both the objective correction collar and the deformable mirror with a bead sample. There was a slight difference in thickness between the sample coverslip (∼0.17mm) and the cranial window (2×0.1mm) ; the real refractive index of the cortical tissue is often simulated to be 1.36 [3], slightly higher than water, which is close to 1.32 for 1300nm light [23]. For this reason and the practically constrained NA, we did not expect, nor did we observe, that additional depth-dependent spherical aberration would have a significant impact during animal imaging. Spherical-related modes $\left(Z_{4}^{0},B_{4}^{0}\right)$ were thus excluded in the animal imaging experiments to ensure a consistent comparison, as both $Z_{4}^{0}$ and $B_{4}^{0}$ , to some different extent, introduce axial focal shift when applied to a truncated Gaussian illumination (see Supplement 1 Figure S2). There are established practices to remove defocus from spherical modes [24] by modifying the modal definitions. For the scope of this paper, we decided to use the original definitions of Zernike and w-Bessel modes without modifications for a fair comparison.

At 850μm depth, both w-Bessel modes and Zernike modes achieved more than twice the improvement in the standard deviation (STD) of red and green channel pixel values, with w-Bessel modes providing a slightly higher image quality. (Figure 3(c)) The image after correction with w-Bessel modes also appeared to be brighter and sharper compared to the Zernike modes corrected image (Fig. 3(a), b). Correction using w-Bessel modes converged rapidly, with most modal coefficients approaching zero after the first correction iteration and remaining stable in subsequent iterations (Fig. 3(d)). In contrast, Zernike modes showed substantial coefficient fluctuations across all three iterations, particularly for coma (dashed boxes), indicating unstable convergence. Consistent with this observation, the wavefronts constructed after Zernike correction changed significantly in shape through iterations, but w-Bessel resultant wavefronts retained similar shapes throughout. The final Zernike-corrected wavefront exhibited larger peak-to-valley amplitudes than the w-Bessel solution (Fig. 3(e)). Despite these differences in wavefront structure, the reconstructed Gaussian-weighted complex fields showed similar features near the beam centre for both approaches, with discrepancies primarily confined to the pupil edges where the illumination intensity is low.

At 1300μm depth, the advantages of w-Bessel modes became more pronounced. Representative images showed stronger signal recovery and improved contrast (higher maximal pixel values) compared to both system-flat and Zernike-corrected conditions (Fig. 3(f) and (h)). Line-profile analysis confirmed increased peak intensity for both correction strategies, with w-Bessel modes providing slightly enhanced contrast and reduced spatial distortions (Fig. 3 g). Notably, Zernike correction introduced a lateral shift of the field of view due to the introduced coma, whereas w-Bessel correction preserved spatial alignment (Fig. 3(f), insets).

Overall, w-Bessel modes enabled faster and more stable sensorless AO convergence, thereby requiring fewer acquisitions for effective correction. This improved robustness makes them particularly well-suited for practical in vivo three-photon imaging, where stability and acquisition efficiency are critical. Additional examples from different animals are provided in Supplement 1 Figure S4.

## 4. Conclusion

Modal-based adaptive optics has traditionally relied on fixed sets of aberration modes applied under fixed illumination conditions, chosen primarily for convenience or historical precedent rather than for optimal performance across imaging depths. In this work, we have addressed the limitations of this approach by introducing an adaptive strategy in which both the illumination beam profile and the aberration correction basis are tailored to the imaging depth and focal position. To our knowledge, this is the first study to theoretically derive and experimentally validate depth-adapted modal bases optimised for deep-tissue three-photon imaging.

Our results demonstrated that adaptable control of the illumination beam profile is essential for efficient fluorescence generation under power-limited conditions. In the mouse cortex, the optimal objective underfilling ratio decreased systematically with depth, shifting from β≈0.73 at 600μm to β≈0.58 at 1300μm . This depth-dependent underfilling substantially improved signal generation at depth and highlighted the limitations of fixed illumination strategies commonly used in three-photon microscopy. While we maintained a constant average laser power for a fair comparison, which is reasonable to account for thermal damage, the laser-induced nonlinear tissue damage was ignored [22]. The Gaussian illuminatio vn has a higher excitation power density at the centre. The current experiments avoided tissue damage, but we would expect a slightly different relationship if the power density is limited, instead of the total average power, to avoid laser-induced nonlinear damage in deep tissue.

Crucially, this adaptive illumination strategy necessitated a corresponding change in the aberration correction basis. We showed that conventional Zernike modes, while widely used, lose orthogonality under truncated Gaussian illumination, leading to slow convergence, unstable correction and resulting in excessive wavefront deformations (with a risk of saturating deformable mirror actuators) and field-of-view shifts. In contrast, the w-Bessel modes introduced here preserve orthogonality under underfilled illumination and enable fast, stable, and reliable sensorless aberration correction. These advantages arise from the intrinsic orthogonality of the w-Bessel basis and are therefore expected to extend beyond the specific indirect AO implementation used in this study. For example, these conclusions are not confined to 3-P microscopy, but would be relevant for any non-linear microscope – including two-photon or harmonic generation – where use of underfilled illumination pupils is advantageous.

This proof-of-principle demonstration showed the benefit of w-Bessel modes over Zernike modes for low order aberration correction. We can consider how this would extend to higher orders of correction. We would expect to see a stronger consequence of non-orthogonality of Zernike modes with more modes present in the correction process. This is because higher order Zernike modes tend to have phase variations even more concentrated near the edge of the pupil, where the amplitude is low. One might consider using the Gaussian Zernike modes, a set of modified Zernike modes to restore the orthogonality for a Gaussian illumination. However, compared to Zernike modes, Gaussian Zernike modes result in even weaker central correction power and even larger phase gradients at the edge of the pupil. These phase gradients make control of the deformable mirror challenging. The disadvantage of this effect is not just a large coefficient and hence more difficulty for convergence, but also leads to readily saturated actuators. Hence, we maintain that Gaussian Zernike modes, despite their orthogonality property, are not suitable for practical aberration correction methods in microscopy.

Together, these results establish a general and practical strategy for deep three-photon microscopy, combining adaptive beam shaping with an illumination-matched aberration basis for AO correction. This unified approach enables stable, efficient aberration correction under realistic power constraints and provides a robust foundation for extending high-resolution in vivo imaging to greater depths.

## Supplemental information

Supplement 1 Supplemental document https://doi.org/10.6084/m9.figshare.33196554

## Data Availability

Data underlying the results presented in this paper are not publicly available at this time but may be obtained from the authors upon reasonable request.

## Supplemental document

See Supplement 1 for supporting content.
