# Supplementary material for: Depth-adapted adaptive optics for three-photon microscopy
Source: Biomed Opt Express. 2026 Aug 13;17(9):4605–16. doi: 10.1364/BOE.609127 (PMC13592269; doi:10.1364/BOE.609127)
Supplement: Supplementary file 1 [file boe-17-9-4605-s001.pdf]

Supplemental Document

Biomedical Optics EXPRESS

Depth-adapted adaptive optics for three-photon microscopy: supplement

QI HU,[1,†] JINGYU WANG,[1,†] HURIYE ATILGAN,[2,†] ARMIN LAK,[2] AND MARTIN J. BOOTH[1,*]

[1] *Department of Engineering Science, University of Oxford, Oxford, UK*
[2] *Department of Physiology, Anatomy, and Genetics, University of Oxford, Oxford, UK*
[†] *These authors contributed equally.*
[*] *martin.booth@eng.ox.ac.uk*

This supplement published with Optica Publishing Group on 13 August 2026 by The Authors under the terms of the Creative Commons Attribution 4.0 License in the format provided by the authors and unedited. Further distribution of this work must maintain attribution to the author(s) and the published article's title, journal citation, and DOI.

Supplement DOI: https://doi.org/10.6084/m9.figshare.33196554

Parent Article DOI: https://doi.org/10.1364/BOE.609127

DEPTH-ADAPTED ADAPTIVE OPTICS FOR THREE-PHOTON MICROSCOPY: SUPPLEMENTAL DOCUMENT

1. Depth-optimised illumination beam size: numerical analysis

The beam size optimisation analysis was adapted from the work [1]. We assumed a rotationally symmetrical Gaussian incident field expressed as

$$E(r,\beta) = \mathrm{N}\exp\left(-\frac{r^2}{\beta^2 R^2}\right)$$

where N is a normalisation term defined below, r is the radial coordinate of the incident plane, β is the underfilling ratio between the radius of the Gaussian beam and the radius of the objective back aperture and R is the radius of the objective back aperture. If $\beta = 1$, the resultant beam is a truncated Gaussian with a field amplitude at the objective aperture equal to e^{-1}, and intensity equal to e^{-2} (or about 13.5%) of the intensity at the centre.

The incident beam is normalised to ensure the total intensity of the incident beam is kept constant while adjusting β. When integrating the intensity of the Gaussian field between $r = 0$ to $r = R$, the expression can be approximated as

$$\int_0^R E^2(r,\beta)\, r\, dr = \left[C\exp\left(-2\frac{r^2}{\beta^2 R^2}\right)\right]_0^R = C\left(1-\exp\left(-\frac{2}{\beta^2}\right)\right)$$

where C is an integration constant. The normalisation term, N, to the total Gaussian incident field integrating over the circular aperture with respect to the field with $\beta = 1$ is thus written as

$$N = \frac{1}{\beta}\sqrt{\frac{1-\exp(-2)}{1-\exp\left(-\frac{2}{\beta^2}\right)}}$$

The incident field can also be expressed in the spherical coordinates of the ray model, written as

$$E(\alpha,\beta) = \mathrm{N}\exp\left(-\frac{\sin^2\alpha}{\beta^2\sin^2\alpha_{\max}}\right)$$

where α_{max} is the largest angle of ray incidence, which relates to the NA of the objective lens in the propagation medium. α is the angle of ray incidence. The spherical coordinates expression helps to derive the attenuation term as the light propagates to a distance in tissue.

Light gets attenuated based on the propagation distance (which relates to the imaging depth, l_{depth}), and the attenuation depth of the medium, l_e, with a relation

$$atten = \exp\left(-\frac{l_{depth}}{2l_e\cos\alpha}\right)$$

Putting the normalisation, Gaussian field and attenuation term together, we can write the incident Gaussian field as

$$E_{gauss}^{inc}(\alpha,\beta) = \frac{1}{\beta}\sqrt{\frac{1-\exp(-2)}{1-\exp\left(-\frac{2}{\beta^2}\right)}}\exp\left(-\frac{\sin^2\alpha}{\beta^2\sin^2\alpha_{max}}\right)\exp\left(-\frac{l_{depth}}{2l_e\cos\alpha}\right)$$

In our analysis, α_{max}s determined by the numerical aperture of the objective lens. l_e was the attenuation depth in the mouse cortex and immersion water for our selected illumination wavelength, where we used $l_e = 293\ \mu m$ in the cortex and $l_e = 10\ mm$ in water [2, 3].The working distance of our objective lens was $4\ mm$.

The vectorial electric field model for a high NA objective lens' focal volume at point P (initially presented by Richards and Wolf in [4]) with linear-x-polarised light can be written as (after removing some constant multipliers)

$$E_x(P) = I_0 + I_2\cos 2\phi_P$$
$$E_y(P) = I_2\sin 2\phi_P$$
$$E_z(P) = -2iI_1\sin\phi_P$$

where ϕ_P is the angular co-ordinate of point P on the lateral focal plane. i is the imaginary unit. After removing any constant, I_0, I_1 and I_2 at point P can be written as

$$I_0(P) = \int_0^{\alpha_{max}} E_{gauss}^{inc}(\alpha,\beta)\sqrt{\cos\alpha}\sin\alpha\,(1+\cos\alpha)J_0(k\rho\sin\alpha)\exp(ikz\cos\alpha)\,d\alpha$$
$$I_1(P) = \int_0^{\alpha_{max}} E_{gauss}^{inc}(\alpha,\beta)\sqrt{\cos\alpha}\sin^2\alpha\,J_1(k\rho\sin\alpha)\exp(ikz\cos\alpha)\,d\alpha$$
$$I_2(P) = \int_0^{\alpha_{max}} E_{gauss}^{inc}(\alpha,\beta)\sqrt{\cos\alpha}\sin\alpha\,(1-\cos\alpha)J_2(k\rho\sin\alpha)\exp(ikz\cos\alpha)\,d\alpha$$

where J_m is the mth order of Bessel functions of the first type, ρ is the radial co-ordinate of point P on the lateral focal plane and z is the axial distance from point P to the focal plane.

And finally, the generated total three-photon signal, S_3, for a volume object can be expressed as

$$S_3 = \int_V \left(|E_x(P)|^2 + \left|E_y(P)\right|^2 + |E_z(P)|^2\right)^3 dP$$

where V is the entire focal volume. We calculated numerically the three-photon signal, summing the pixels above the threshold of $1/10^3$ of the maximal value to encompass most of the power of the focus.

For each proposed imaging depth in cortex, we computed and optimised the three-photon signal S_3 by varying the filling ratio β. Results are included in the main paper Figure 1.

2. Beam profile measurement

To assess the effect of the illumination underfilling ratio, we characterised the imaging system by measuring the beam profile and power at the objective back aperture while adjusting the beam expander to adjust the beam size, and the half-waveplate to adjust the beam power.

For beam-profile measurements, the beam expander setting was fixed at infinite conjugate to ensure a collimated output beam. The expansion ratio was varied from $0.5\times$ to $2.5\times$. The beam profile was measured using a power meter with a circular aperture of $9.5mm$ (Thorlabs PM100D, SC405C sensor head). To obtain the intensity distribution, black tape was applied to the power-meter head, leaving a narrow central slit ($\sim 1mm$) exposed. The taped power meter

was mounted near the objective back aperture and translated laterally in 1 mm steps; power was recorded at each position.

The measured slit-integrated power was used to reconstruct the beam profile by assuming a rotationally symmetric Gaussian shape, consistent with the manufacturer's manual and visual inspection using an IR card and viewer. In addition, a point-based 2-D beam scan was also used to verify the Gaussian beam size and beam shape. A The underfilling ratio, β, was calculated as the ratio of the beam diameter (cut-off at 13.5% of peak intensity) to the objective aperture diameter.

Beam power after the objective was also measured for each expansion setting while adjusting the half-waveplate, ensuring fair comparison at constant illumination power across imaging depths. This calibration process was performed once, and the beam expander was found to increase beam size linearly and consistently within measurement uncertainty.

3. Three-photon fluorescence microscopy

Three-photon fluorescence imaging was carried out using a Cronus 3P laser system (Light Conversion), which integrates a tuneable optical parametric amplifier and prism compressor, powered by a 1030 nm femtosecond laser. The setup was optimized for 1300 nm excitation and delivered 49 fs pulses (measured under the objective using an APE autocorrelator with an external detector) with a 1 MHz repetition rate. During animal experiments, the laser power was adjusted to 50 mW at the sample. Image acquisition was conducted using a galvanometer-based multiphoton system (VivoScope, Scientifica, UK) equipped with a water-immersion objective (Olympus, XLPLN25XSVMP2, 25×/1.0 NA, 4 mm WD).

Green and red fluorescence signals were detected via separate PMT channels (Hamamatsu H10770PA-40) using dichroic (T565LPXR, Chroma) and bandpass filters (ET525/50m-2p for green, ET620/60m for red, Chroma). The signals were processed through a 150 kOhm, 1 MHz transimpedance amplifier (XPG-ADC-PREAMP) and digitized using a vDAQ card (Vidrio Technologies). ScanImage 2021 software (Vidrio Technologies), running on MATLAB, managed the image acquisition. The system included a motorized stage and focusing unit to control sample and objective positioning. Imaging depths were measured via axial displacement of the stage. For most structural scans, we used a 256×256 resolution with 3.2 µs dwell time at 4.23 Hz, averaging across 240 frames unless noted otherwise.

4. Adaptive optics and aberration correction

4.1 Deformable mirror (DM) calibration

Prior to usage, the continuous-membrane DM underwent a complete Zernike mode calibration using a custom-built interferometer [5]. Each actuator was systematically driven across voltages while interferograms were captured. These were processed via custom Python software (https://github.com/dop-oxford/dmlib) to extract wavefronts, fit actuator responses to Zernike basis functions, and generate a mode-to-voltage transformation matrix. This ensured accurate rendering of any target wavefront correction during imaging.

4.2 Optical relay path

To compensate for optical distortions during deep tissue imaging, one of two DMs (Imagine Optic Mirao 52e or Alpao DM97) was alternatively introduced into the beam path. The beam, expanded post-OPA to match the DM's aperture (15 mm and 13.5 mm respectively), was directed through a telescope system that aligned the DM with both the scanning mirrors and the back pupil plane of the objective, ensuring uniform correction across the field of view, via two

relay-lens pairs of 500/60 mm and 30/206 mm focal lengths (not shown in Figure 1a). A secondary 8.3× beam reduction with a telescope with lenses of 500 mm and 60 mm focal lengths followed, bringing the beam size to 1.8 mm (1.62 mm for DM97) at the Galvo scanners, which was then re-expanded at the objective pupil to 12.75 mm (11.57 mm for DM97), resulting in a diameter fill ratio (DM aperture to objective aperture) of 0.85.

4.3 Sensorless AO correction

During imaging experiments, we used a sensorless AO approach through which image quality metrics—such as brightness or contrast—served as feedback to guide correction. We iteratively applied varying amplitudes of specific Zernike modes and measured image quality metrics to identify the optimal correction. Tip, tilt, and defocus modes were excluded to avoid field-of-view displacement. The remaining modes were optimized one at a time by fitting a parabola or Gaussian curve to metric values across five evenly spaced bias RMS amplitudes within the range of ±1.5 or ±3 rad (as stated in each set of results). This Gaussian fitting enabled estimation of the best correction within the selected bias range for each aberration.

We employed the 5N correction scheme, applying five biases per mode. For animal corrections, primary astigmatism, coma and trefoil in Zernike (Z_2^(±2), Z_3^(±1), Z_3^(±3)) and their corresponding w-Bessel modes (B_2^(±2), B_3^(±1), B_3^(±3)) were included. Though originally demonstrated on two-photon systems [6], the methodology extends naturally to three-photon excitation [7].

For system flat DM pattern generation, the objective correction collar was first adjusted to compensate for coverslip-induced spherical aberration. Based on the DM flat pattern, the system flat DM pattern was measured from a beads sample using standard sensorless AO algorithm when the beam was adjusted to $\beta \approx 0.72$, which served as a starting point for subsequent correction. Seven Zernike modes (Z_2^(±2), Z_3^(±1), Z_3^(±3), Z_4^0) were included in the correction.

5. Animal preparation

All animal work adhered to the UK Animals (Scientific Procedures) Act 1986. Experiments were carried out using adult C57BL/6 mice, including both males and females, aged between 2 and 6 months. The study included three genetically modified mice from the CamKIIα–tTa (AI94) × B6; DBA-Tg(tetO-GCaMP) line, with animals expressing GCaMP8s. Mice were housed in controlled conditions, maintained at 20–22 °C with 40% relative humidity, on a standard 12-hour light/dark cycle. Animals had unrestricted access to food, and their health and wellbeing were monitored daily.

Animal surgeries were performed following the same protocol described in [8]. Briefly, anaesthesia was induced with 3% isoflurane and maintained at or below 1.5% throughout the procedure. Mice received perioperative analgesia with subcutaneous buprenorphine and meloxicam, and the scalp was locally treated with bupivacaine prior to sterilisation and incision. After cleaning the skull and attaching a custom aluminium head-plate, a circular craniotomy (4–5 mm diameter) targeting the prelimbic cortex was performed. For animals receiving viral labelling, a retrograde virus (rAAV2-CAG-tdTomato) was injected into the dorsomedial striatum (DMS) using a glass pipette and nanoinjector, followed by durotomy and cranial window implantation with a double coverslip. Mice were allowed to recover in a heated chamber and monitored for 7 days, with a minimum recovery period of 21 days prior to behavioural experiments to allow for viral expression.

For the beads slide, 0.5 μm red fluorescent beads (Invitrogen™ # F8812) were diluted 100 times before being laid on standard #1.5 microscope coverslip to dry. VECTASHIELD Antifade mounting medium (Vector Laboratories, UK) was used to mount the beads-coated coverslips on microscope slides. They were then sealed with nail varnish.

6. Modal comparison and analysis

To further examine the stability of different aberration bases, we compared the effects of the Zernike coma modes ($Z_3^{\pm1}$) and their weighted-Bessel counterparts ($B_3^{\pm1}$) using fluorescent bead samples (Supplementary Figure S1). When applied over a range of phase amplitudes, the Zernike coma modes produced substantial modulation of fluorescence intensity, accompanied by noticeable lateral shifts in the bead centre of gravity in the x–z plane. In contrast, the corresponding w-Bessel modes yielded comparable intensity modulation while substantially reducing lateral displacement, indicating improved decoupling between aberration correction and beam translations.

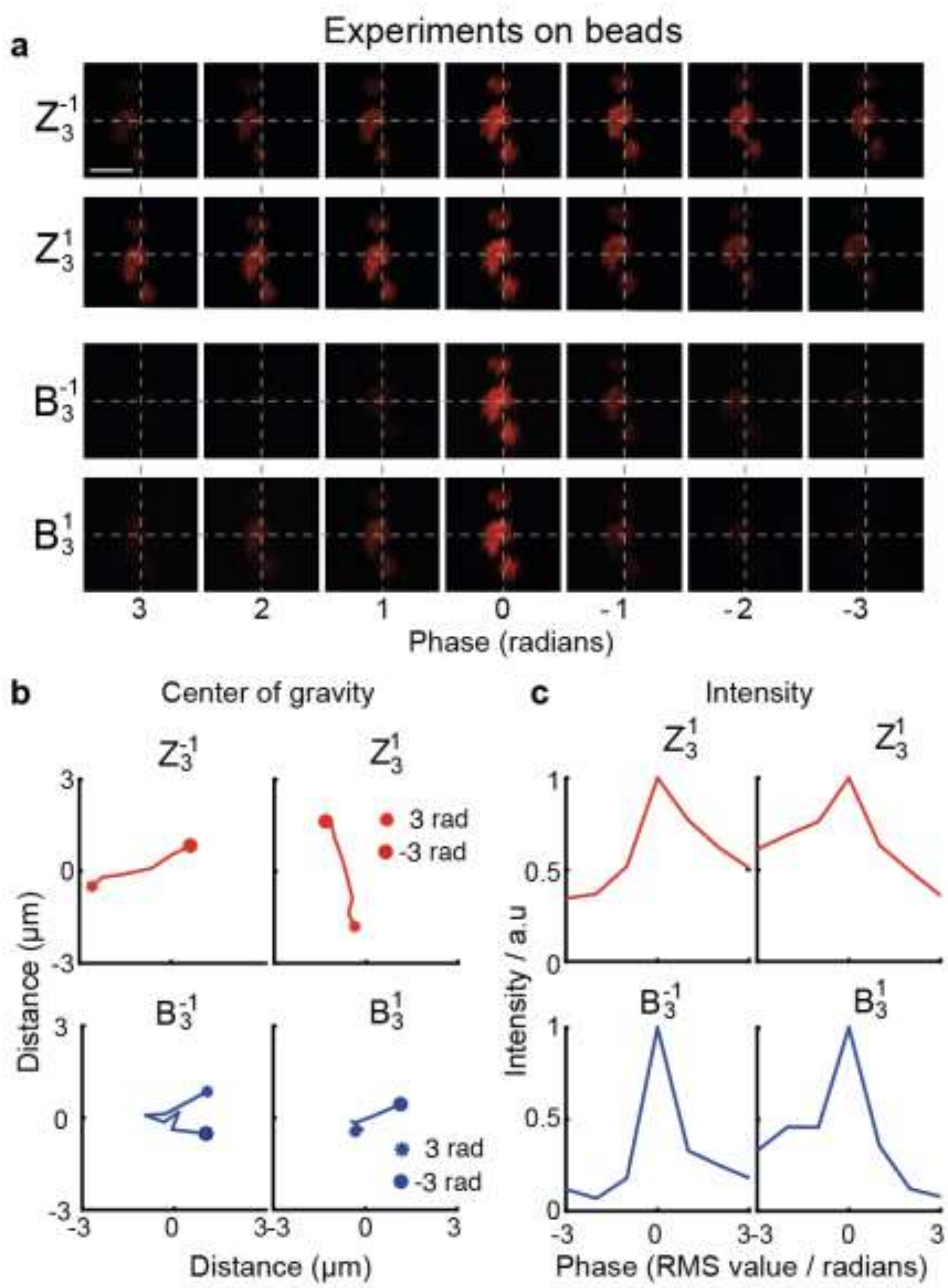

Fig. S1. **Field of view shifts induced by Zernike but not by weighted-Bessel aberration modes.** (a) x–z bead images acquired while applying Zernike and weighted-Bessel modes across phase amplitudes from −3 to +3 rad. (b) Lateral centre-of-gravity displacement as a

function of phase for Zernike (red) and weighted-Bessel (blue) modes. (c) Normalised fluorescence intensity as a function of phase for the same modes.

For comparison, we also examined the effect of the Zernike spherical mode (Z_0^4) and its corresponding w-Bessel mode (B_0^4) in bead measurements (Supplementary Figure 2). In this case, the applied spherical aberration resulted in relatively weak intensity modulation but induced a pronounced axial shift of the focal plane. This behaviour reflects strong cross-talk between spherical aberration and defocus under truncated Gaussian illumination, consistent with the non-orthogonality observed in the Zernike basis. Together, these results highlight that w-Bessel modes provide improved control of aberrations while minimising unintended focal shifts, which is critical for stable sensorless adaptive optics correction.

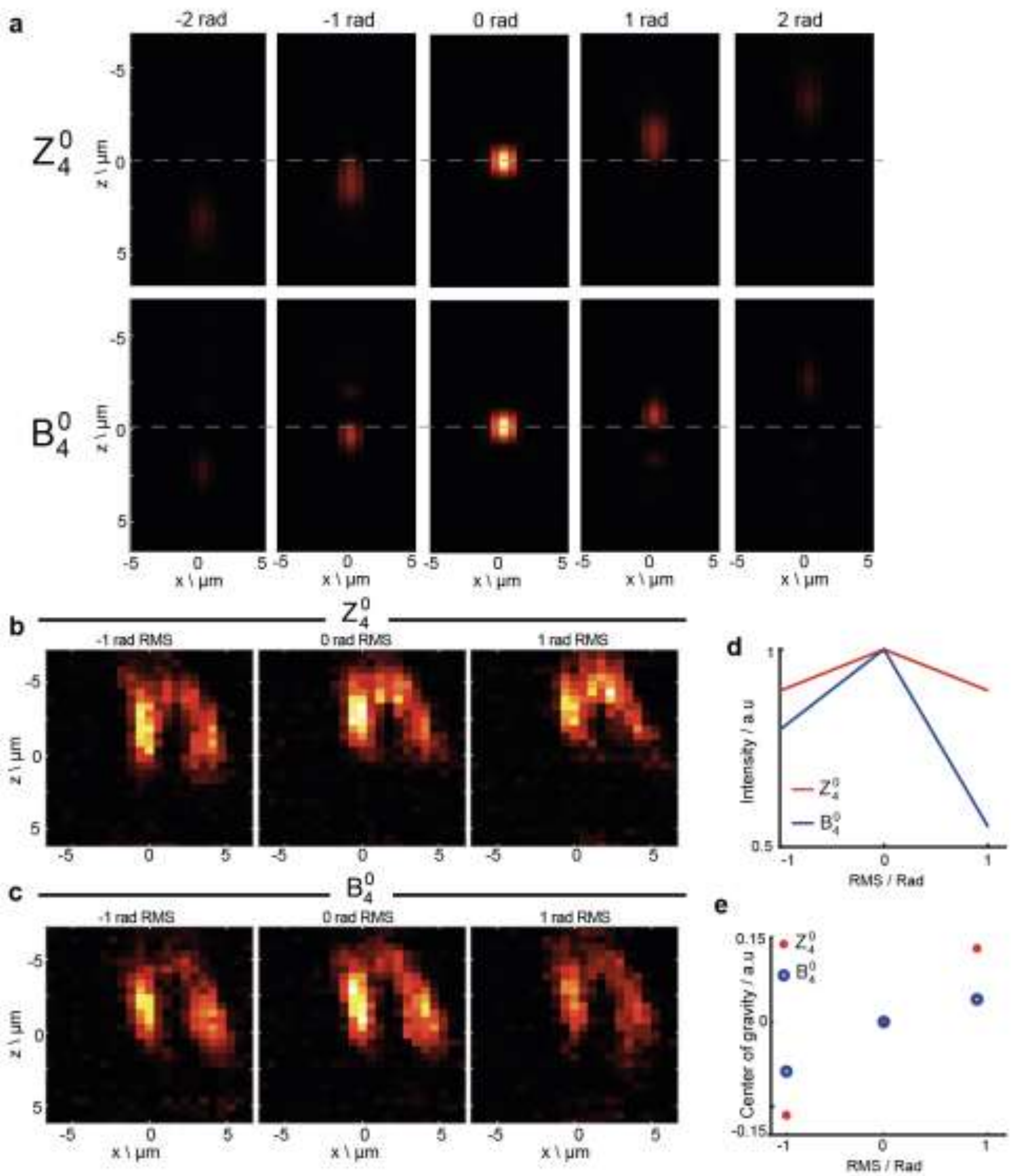

Fig. S2. **Differential effects of Zernike and weighted-Bessel spherical modes on focus quality and axial stability.** (a) Simulation for Zernike and Bessel spherical aberration modes. (b) x–z plane images of a fluorescent bead sample obtained while applying the Zernike spherical aberration mode Z_4^0 with amplitudes of −1, 0, and +1 rad RMS (left to right). (c) Weighted-Bessel B_4^0 mode applied at −1, 0, and +1 rad RMS. (d) Corresponding normalised fluorescence intensity as a function of applied RMS phase for Z_4^0 (red) and weighted-Bessel B_4^0

(blue) modes. (e) Corresponding axial centre-of-gravity shifts of the bead signal as a function of applied RMS phase for Zernike Z_4^0 (red) and weighted-Bessel B_4^0 (blue).

7. Additional experimental results on beam expansion effects

Additional experimental results demonstrating the effects of beam expansion on imaging quality at different cortical depths are shown in Supplementary Figure 3. Similar depth-dependent trends were observed across multiple animals and imaging sessions, and the optimal filling ratios were consistent across different illumination power levels, indicating that the effect was robust and not dependent on a specific preparation or power regime.

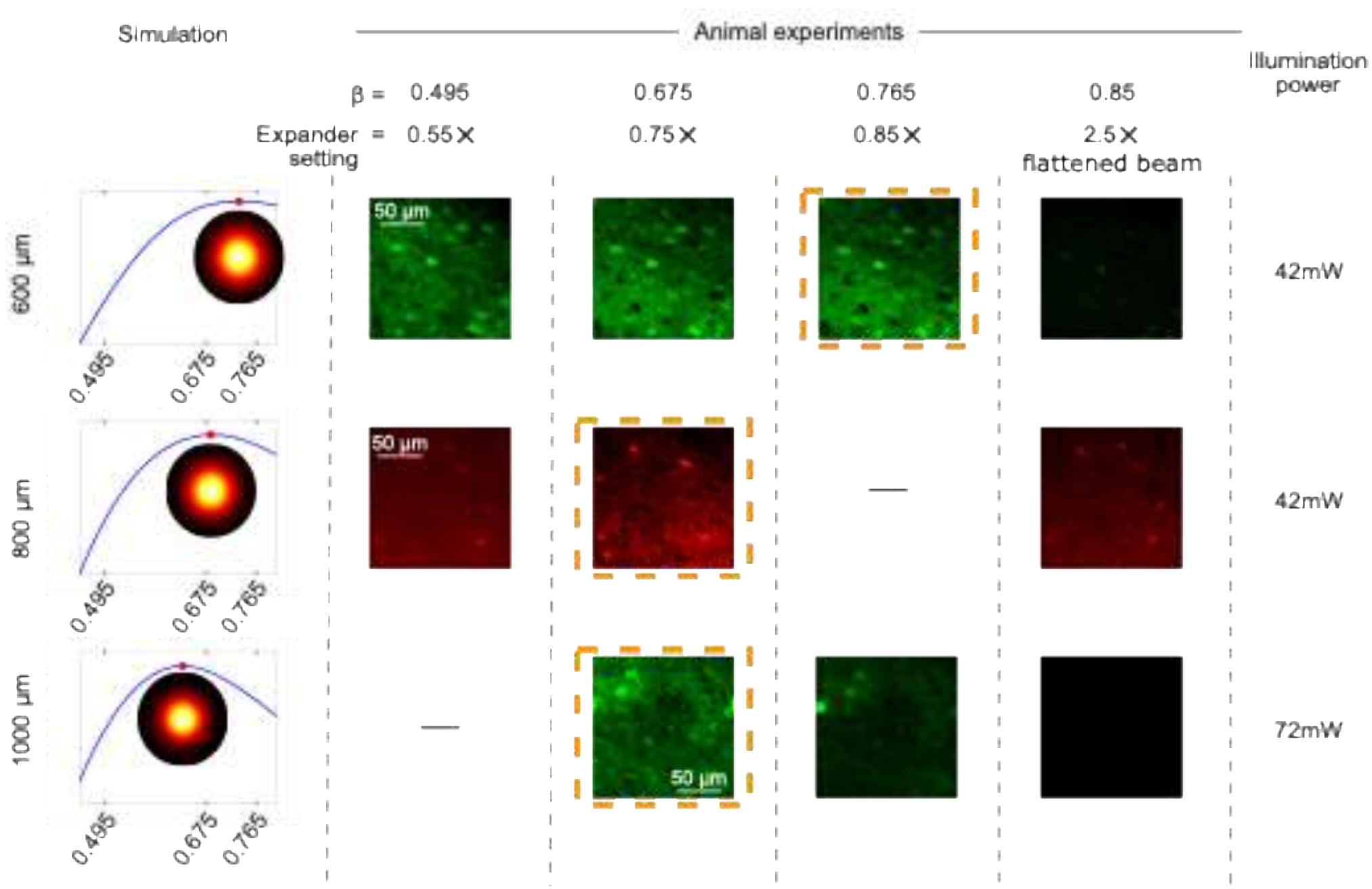

Fig. S3. **Depth-dependent optimisation of illumination beam expansion.** Simulation of three-photon signal as a function of illumination filling ratio (β) at 600, 800, and 1000 μm depth, with corresponding in vivo images acquired using different beam-expander settings and a flattened beam profile. At each depth, the brightest images correspond to the β values predicted by simulation.

8. Experimental results on modal comparison

Additional experimental results comparing Zernike modes and w-Bessel modes were included in supplementary Figure S4. Two experiments at 900 μm and 1000 μm depth was shown. W-Bessel modes showed consistently superior corrections as evidenced through the resultant images being brighter and sharper, with a larger number of high pixel values. The correction also converged faster, where w-Bessel generally optimised after the first iteration, and Zernike modes had coefficients fluctuating even after three iterations of correction.

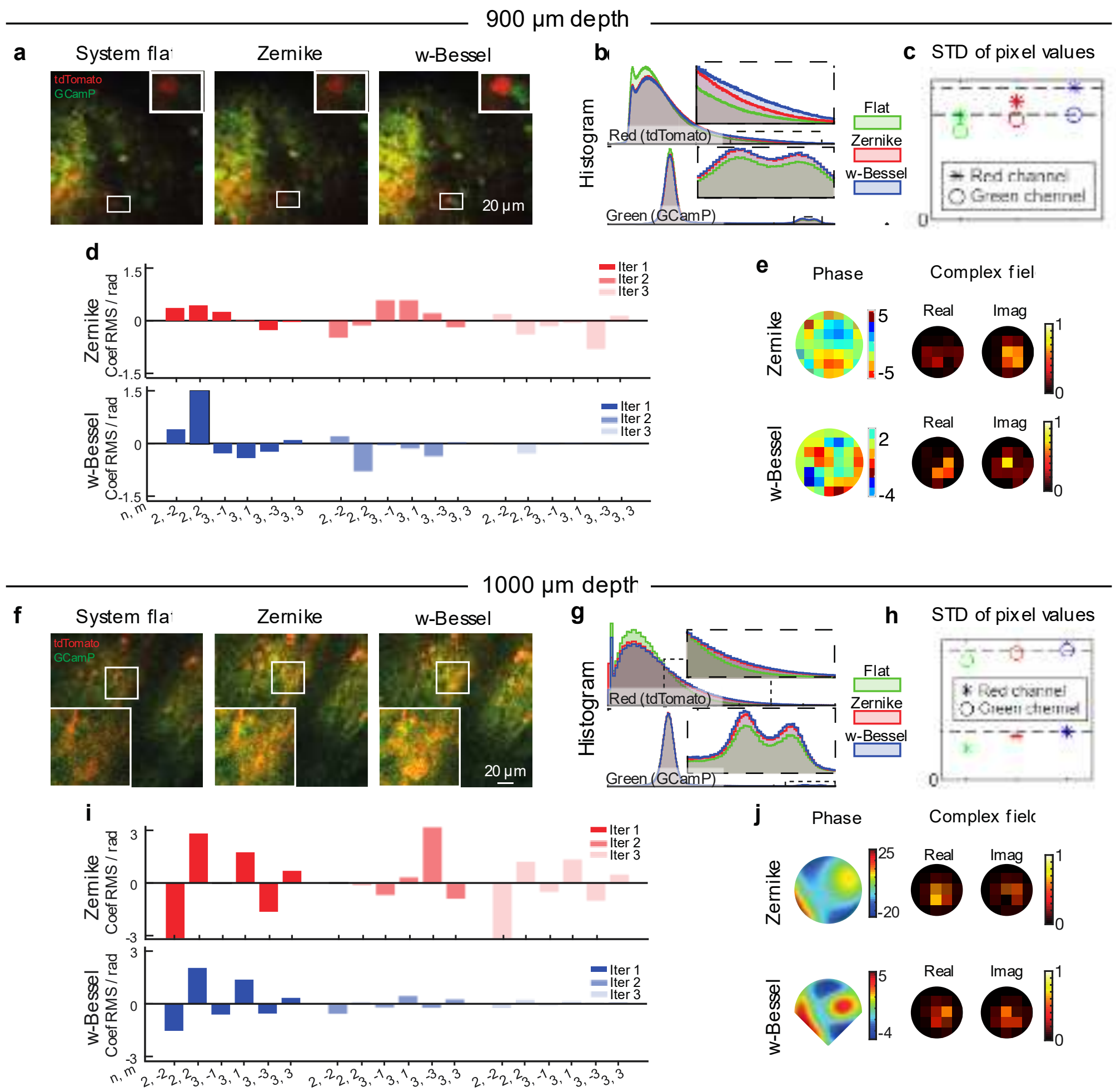

Fig. S4. **Comparisons of w-Bessel and Zernike mode based correction of aberrations** at (a-e) 900 μm and (f-j) 1000 μm in an awake mouse cortex for the designed beam profile. (a, f) The selected FOV averaged from 120 frames when the AO was applying a system correction (left image), and after applying corrections generated from the Zernike set (middle image) and w-Bessel set (right image). (b, g) the histogram comparison of the images before and after corrections. (c, h) the standard deviation (STD) of all red and green channel pixel values of images in (a) and (f). (d, i)The bar charts showed the computed modal coefficients after each of the three iterations of corrections using Zernike and w-Bessel modes. (e, j) The corresponding wavefront corrections and the resultant gaussian illumination with real and imaginary intensity components.

9. Image Processing

For quantitative image comparisons (e.g. Figures 1 and 3 in the main paper), image sequences consisting of 240 frames (for two colour channels or 120 frames for a single colour channel of 30 s acquisition at 4 Hz) were corrected for in-plane motion using a NoRMCorre-based motion correction algorithm. The registered frames were subsequently averaged to generate a single representative image for quantitative analysis. Averaging across the acquisition reduced the influence of transient physiological fluctuations and residual frame-to-frame variability while preserving the underlying fluorescence distribution. To avoid systematic bias arising from acquisition order, comparisons between Zernike- and weighted-Bessel-based adaptive optics

corrections were counterbalanced across imaging locations. All image processing was applied identically to both correction strategies

References

1. K. Wang, R. Liang, and P. Qiu, "Fluorescence Signal Generation Optimization by Optimal Filling of the High Numerical Aperture Objective Lens for High-Order Deep-Tissue Multiphoton Fluorescence Microscopy," Ieee Photonics J 7, 1-8 (2015).

2. T. Wang, C. Wu, D. G. Ouzounov, et al., "Quantitative analysis of 1300-nm three-photon calcium imaging in the mouse brain," eLife 9, e53205 (2020).

3. T. Wang, and C. Xu, "Three-photon neuronal imaging in deep mouse brain," Optica 7, 947-960 (2020).

4. B. Richards, and E. Wolf, "Electromagnetic diffraction in optical systems, II. Structure of the image field in an aplanatic system," Proceedings of the Royal Society of London. Series A. Mathematical and Physical Sciences 253, 358-379 (1959).

5. J. Antonello, J. Wang, C. He, et al., "Interferometric calibration of a deformable mirror," Zenodo. 2020b doi 10 (2020).

6. A. Facomprez, E. Beaurepaire, and D. Débarre, "Accuracy of correction in modal sensorless adaptive optics," Optics express 20, 2598-2612 (2012).

7. L. Streich, J. C. Boffi, L. Wang, et al., "High-resolution structural and functional deep brain imaging using adaptive optics three-photon microscopy," Nat Methods 18, 1253-1258 (2021).

8. H. Atilgan, J. Wang, Q. Hu, et al., "Deep three-photon functional imaging in the mouse cortex with adaptive optics," bioRxiv, 2025.2005. 2004.652136 (2025).
